# Empirical Antifungal Treatment of Critically Ⅲ Patients With Influenza-Associated Acute Respiratory Distress Syndrome: A Propensity Score Weighted Observational Study

**DOI:** 10.1093/cid/ciaf507

**Published:** 2025-09-15

**Authors:** Stefan Hatzl, Lisa Kriegl, Christina Geiger, Caroline Wilhelmer, Alexander C Reisinger, Markus Keldorfer, Julia Auinger, Gernot Schilcher, Florian Krammer, Philipp Eller, Robert Krause

**Affiliations:** Department of Internal Medicine, Intensive Care Unit, Medical University of Graz, Graz, Austria; Department of Microbiology, Icahn School of Medicine at Mount Sinai, New York, New York, USA; Center for Vaccine Research and Pandemic Preparedness (C-VaRPP), Icahn School of Medicine at Mount Sinai, New York, New York, USA; BioTechMed Graz, Graz, Austria; BioTechMed Graz, Graz, Austria; Division of Infectious Diseases, Department of Internal Medicine, Medical University of Graz, Graz, Austria; Division of Infectious Diseases, Department of Internal Medicine, Medical University of Graz, Graz, Austria; Department of Internal Medicine, Intensive Care Unit, Medical University of Graz, Graz, Austria; Department of Internal Medicine, Intensive Care Unit, Medical University of Graz, Graz, Austria; Department of Children and Adolescent Medicine, Pediatric Intensive Care Unit, Medical University of Graz, Graz, Austria; Department of Internal Medicine, Intensive Care Unit, Medical University of Graz, Graz, Austria; Department of Internal Medicine, LKH Südsteiermark—Standort Wagna, Wagna, Austria; Department of Microbiology, Icahn School of Medicine at Mount Sinai, New York, New York, USA; Center for Vaccine Research and Pandemic Preparedness (C-VaRPP), Icahn School of Medicine at Mount Sinai, New York, New York, USA; Department of Pathology, Molecular and Cell-Based Medicine, Icahn School of Medicine at Mount Sinai, New York, New York, USA; Ignaz Semmelweis Institute, Interuniversity Institute for Infection Research, Medical University of Vienna, Vienna, Austria; Department of Internal Medicine, Intensive Care Unit, Medical University of Graz, Graz, Austria; BioTechMed Graz, Graz, Austria; Division of Infectious Diseases, Department of Internal Medicine, Medical University of Graz, Graz, Austria

**Keywords:** ICU, IAPA, influenza associated aspergillosis, posaconazole, mould treatment

## Abstract

**Background:**

Influenza-associated pulmonary aspergillosis (IAPA) is a significant fungal complication in patients with influenza-induced acute-respiratory-distress-syndrome (ARDS). The impact of empirical antifungal treatment on IAPA incidence and outcomes remains unclear.

**Methods:**

In this observational multicenter study (9 treatment centers), we included all consecutive patients admitted to intensive care units (ICUs) with influenza-associated ARDS between 1 September 2016 and 1 March 2025. We compared patients receiving empirical antifungal treatment with those who did not, focusing on 30-day IAPA incidence (primary outcome) and survival (secondary outcome). Propensity score weighting was used to account for baseline characteristic imbalances. IAPA cases were classified based on the Fungal-Infections-in-Adult-Patients-in-ICU (FUNDICU) consensus criteria.

**Results:**

We included 172 patients, 61 (35%) of whom received empirical antifungal therapy (94% posaconazole). IAPA was diagnosed in 24 cases, with a median onset of 2 days after ICU admission. Of these, 20 occurred in the non-treatment group and 4 in the empirical treatment group. The 30-day IAPA incidence was 7.7% in the treatment group and 20.4% in the non-treatment group (*P* = .002). The sub-distributional hazard ratio (sHR) for IAPA incidence in the empirical treatment group compared with the non-treatment group was 0.21 (95% CI: 0.10–0.92, *P* = .045). However, there was no significant difference in 30-day ICU survival.

**Conclusions:**

In ICU patients with influenza ARDS, empirical antifungal treatment was associated with significantly reduced IAPA incidence, but this did not translate into improved survival. Randomized controlled trials are warranted to evaluate the efficacy and safety of patients’ specific empirical antifungal treatment with regard to IAPA incidence and outcomes.

## Teaser

In this study of 172 ICU patients with influenza-related ARDS, empirical antifungal treatment was associated with reduced pulmonary aspergillosis incidence (7.1% vs 19.6%, *P* = .002), but did not improve 30-day ICU survival. Randomized trials are needed to confirm efficacy and safety.

## Graphical Abstract

**Graphical Abstract:**
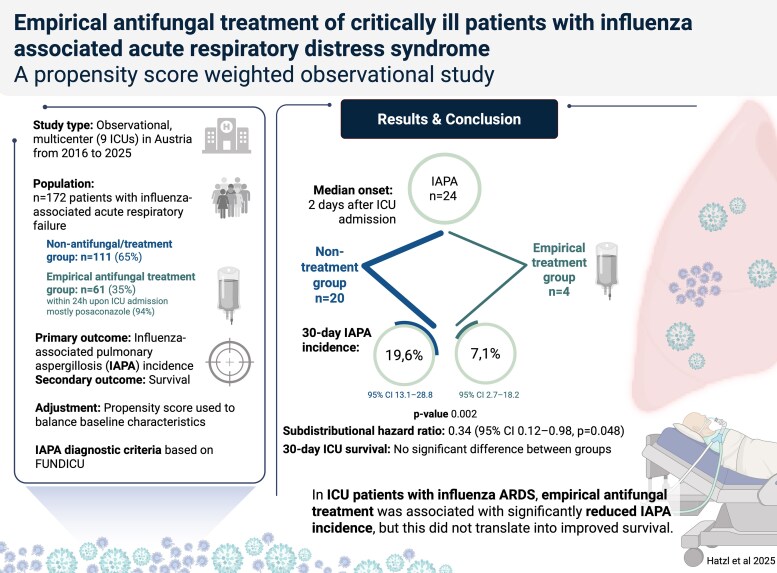

**(See the Editorial Commentary by Reizine and Gangneux on pages e494–6.)**

Worldwide, influenza is a major health threat causing ∼650 000 deaths annually [1, 2]. *Aspergillus* superinfection in critically ill influenza patients was first noted in the 1950s, but only recognized as influenza-associated pulmonary aspergillosis (IAPA) after the 2009 H1N1 pandemic [3–6]. IAPA is a severe complication of influenza, affecting up to 20% of patients admitted to the intensive care unit (ICU) doubling mortality rates to ∼50% in IAPA cases compared with 25% in non-IAPA patients [7]. IAPA is difficult to diagnose due to non-specific imaging (unlike in neutropenic patients), low serum galactomannan sensitivity, and frequent upper airway colonization. Bronchoscopy with bronchoalveolar lavage remains the diagnostic cornerstone, enabling direct lower respiratory tract sampling. In response, updated classification systems now recognize influenza as an independent risk factor for invasive pulmonary aspergillosis in non-neutropenic ICU patients [8–10]. Before the coronarvirus-19 pandemic, efforts were made to prevent IAPA with posaconazole prophylaxis. A previous randomized controlled trial found no significant benefit of posaconazole prophylaxis. However, the study was underpowered, and the 7-day duration of prophylaxis despite remaining risk factors for IAPA may have been too short, as two IAPA cases occurred after cessation of prophylaxis. Additionally, over 70% of IAPA cases emerged within the first 48 h of ICU admission, suggesting that prophylaxis initiated during the ICU stay comes too late [11, 12]. Given the high incidence of IAPA, with most cases present at ICU admission, we aimed to investigate the role of empirical administration of mold active antifungals in critically ill patients with influenza and acute respiratory distress syndrome (ARDS) in a retrospective multicenter observational trial.

## METHODS

### Study Population

We conducted a multicenter retrospective observational study, enrolling all consecutive adult patients with polymerase chain reaction (PCR) confirmed influenza A or B who were admitted to the ICUs of our nine treatment centers for influenza-associated acute respiratory failure between 1 September 2016, and 1 March 2025 (Supplementary Figures 1–3).

Patient data were uniformly collected as previously described, with laboratory, clinical, and radiology data extracted from our in-house electronic healthcare database using REDCap. IAPA classification followed the “Fungal Diseases in Adult ICU Patients” (FUNDICU) algorithm, identifying influenza as the non-neutropenic risk factor [9, 13, 14]. Patients were categorized as having probable or no IAPA, while proven IAPA was diagnosed post-mortem via necropsy in some cases. In all post-mortem confirmed IAPA cases, pathologists identified invasive fungal disease as a major contributor to death.

The study was approved by the local institutional review board (EK: 32-302ex19/20) and conducted in accordance with the Declaration of Helsinki.

### Patients Receiving Empirical Mold-active Antifungals and Control Group

Early empirical administration of mold-active antifungals (ie within the first 24 h after admission) in critically ill patients with influenza and ARDS was based on a local influenza treatment guideline approved by representatives from the ICUs and the division of infectious diseases. This strategy was implemented across all treatment centers for the entire study period. This antifungal strategy was based on local fungal epidemiology with an IAPA case rate exceeding 15% in the past (data not shown). However, execution of the guideline including administration of empirical mold-active antifungals was ultimately at the discretion of the attending ICU physicians. At ICU admission all patients underwent routinely comprehensive evaluation for the presence of an invasive fungal infection (including bronchoalveolar lavage—galactomannan and BAL culture testing for invasive ventilated patients and serum GM testing for non-invasive ventilated patients) to identify early IAPA cases. This enabled us to establish a group of critically ill patients with influenza and ARDS receiving early administration of mold-active antifungals (n = 61) and a control group (n = 111) for analysis.

### Statistical Analysis

All statistical analyses were conducted using Stata (Windows version 18.1; Stata Corp., Houston, TX, USA) and R 4.4.1 (https://www.r-project.org/) following a pre-specified statistical analysis plan (Supplementary Figure 4). Baseline characteristics between the empirical antifungal treatment and non-antifungal groups were assessed using Mann–Whitney *U* tests, χ²-tests, and Fisher's exact tests, as appropriate. The magnitude of differences between groups was quantified using standardized mean differences (SMDs), with SMDs ≥0.30 considered indicative of a relevant difference. To adjust for covariate imbalances, we performed propensity score analysis (Supplementary Methods) [15].

The primary outcome, 30-day IAPA incidence, was defined as the time from ICU admission to the first positive IAPA sample or censoring at 30 days. For patients who died on the day of ICU admission (n = 4), survival time was adjusted by adding one day.

The secondary outcome was 30-day ICU overall survival (OS), defined as time from ICU admission to death from any cause or censoring at 30 days. We also analyzed 90-day ICU OS to assess long-term outcomes (Supplementary Methods).

## RESULTS

### Cohort Description

A total of 172 patients were admitted to our ICUs with influenza-associated acute respiratory failure and included in the final analysis (Supplementary Figure 2). Of the cohort, 61 patients (35%) received empirical mold-active antifungals within 24 h of ICU admission, while 111 (65%) did not. The median age at ICU admission was 65 years [IQR: 56–73], and 66 patients (38%) were female. Patients had a median of two comorbidities [IQR: 1–4]. Most had a positive influenza PCR on the day of ICU admission, with a median time of 0 [0–1] days between testing and admission. Influenza A virus was detected in 151 patients (88%), and influenza B virus in 21 (12%). All patients presented with oxygen saturation <90% on ambient air and were diagnosed with acute respiratory distress syndrome. Respiratory impairment was severe, with a median PaO₂/FiO₂ ratio of 118 [86–160]. Based on 2024 ARDS guidelines, 74 patients (43%) met criteria for severe ARDS, and 98 (57%) for moderate ARDS [16 ]. Invasive mechanical ventilation was required in 74 patients (43%), and 20 (12%) received veno-venous ECMO. Oseltamivir was administered to 168 patients (98%) following influenza diagnosis (Table 1).

**Table 1.** Data are Reported as Medians [25th–75th Percentile] or as Absolute Counts (%)

Variable	n (%miss)	Overall (n = 172)	Early Empirical Antifungal Treatment (n = 61)	No Early Empirical Antifungal Treatment (n = 111)	*P*	*p_IPTW_*
Demographic variables						
Age (y)	172 (0%)	65 [56–73]	61 [50–72]	66 [58–74]	.**032**	0.941
Female Gender	172 (0%)	66 (38%)	23 (37%)	43 (39%)	.894	0.649
BMI (kg/m²)	172 (0%)	26.4 [23.4–29.4]	24.7 [22.3–28.7]	26.7 [24.3–29.4]	.053	0.786
Influenza Type						
Influenza A	172 (0%)	151 (88%)	56 (92%)	95 (85%)	.231	0.298
Influenza B	172 (0%)	21 (12%)	5 (8%)	16 (14%)	…	…
Coexisting conditions						
Number of coexisting conditions	172 (0%)	2 [1–4]	1 [1–4]	2 [1–4]	.595	0.719
Hypertension	172 (0%)	88 (51%)	26 (43%)	62 (56%)	.100	0.495
Diabetes	172 (0%)	43 (25%)	18 (29%)	25 (22%)	.311	0.125
Atrial fibrillation	172 (0%)	38 (22%)	8 (13%)	30 (27%)	.035	0.244
Coronary artery disease^a^	172 (0%)	44 (26%)	13 (21%)	31 (28%)	.341	0.282
Congestive heart failure	172 (0%)	53 (31%)	16 (26%)	37 (33%)	.334	0.898
Peripheral arterial disease	172 (0%)	9 (5%)	4 (6%)	5 (5%)	.563	0.361
Thromboembolic disease	172 (0%)	19 (11%)	6 (10%)	13 (12%)	.707	0.621
Chronic kidney disease	172 (0%)	33 (19%)	11 (18%)	22 (20%)	.776	0.706
Dialysis	172 (0%)	12 (7%)	5 (8%)	7 (6%)	.642	0.702
COPD	172 (0%)	48 (28%)	16 (26%)	32 (29%)	.716	0.701
Asthma	172 (0%)	5 (3%)	1 (2%)	4 (4%)	.463	0.249
Prior cancer in complete remission	172 (0%)	6 (3%)	1 (2%)	5 (5%)	.327	0.123
Active malignancy	172 (0%)	22 (13%)	13 (21%)	9 (8%)	.**013**	0.956
Prior transplantation	172 (0%)	7 (4%)	3 (5%)	4 (4%)	.676	0.302
Immunosuppression^b^	172 (0%)	26 (15%)	16 (26%)	10 (9%)	.**003**	0.841
ICU risk stratification						
SOFA (points)	172 (0%)	5 [4–7]	5 [4–7]	5 [4–7]	.902	0.797
paO_2_/FiO_2_	172 (0%)	118 [86–160]	120 [92–145]	114 [80–168]	.687	0.766
PEEP (mmHg)—maximum	172 (0%)	9 [8–12]	9 [8–12]	8 [7–12]	.586	0.498
Acute respiratory distress syndrome grading	172 (0%)	…	…	…	.470	0.315
severe	…	74 (43%)	24 (39%)	50 (45%)	…	…
moderate	…	98 (57%)	37 (61%)	61 (55%)	…	…
Ventilation	172 (0%)	…	…	…	.466	0.503
vvECMO	…	20 (12%)	4 (7%)	16 (14%)	…	…
Intubated	…	74 (43%)	29 (48%)	45 (41%)	…	…
NIV	…	75 (44%)	27 (44%)	48 (43%)	…	…
HFNC	…	3 (2%)	1 (2%)	2 (2%)	…	…
Any invasive ventilation	172 (0%)	94 (55%)	33 (54%)	61 (55%)	.914	0.802
Antifungals						
Duration form diagnosis to initiation (d)	172 (0%)	…	0 [0–0]	…	…	…
Antifungal drug used	172 (0%)	…	…	…	…	…
Posaconazole	…	…	53 (87%)	…	…	…
Voriconazole	…	…	4 (7%)	…	…	…
Isavuconazole	…	…	2 (3%)	…	…	…
Caspofungin	…	…	2 (3%)	…	…	…
Laboratory values						
Lactate (mmol/L)	165 (4%)	1.5 [0.9–2.3]	1.3 [0.9–2.3]	1.6 [0.9–2.3]	.197	0.703
IL-6 (pg/mL)	51 (70%)	178 [75–1780]	155 [78–1306]	303 [38–5000]	.687	0.386
CRP (mg/L)	172 (0%)	106 [46–219]	108 [61–196]	105 [41–219]	.666	0.351
Ferritin (ng/mL)	69 (60%)	621 [256–1879]	789 [316–1843]	446 [242–1953]	.442	0.781
Creatinine (mg/dL)	172 (0%)	1.14 [0.84–1.91]	1.04 [0.75–1.42]	1.26 [0.89–2.10]	.**024**	0.374
Bilirubin total (mg/dL)	172 (0%)	0.45 [0.28–0.85]	0.40 [0.25–0.72]	0.51 [0.30–0.88]	.057	0.562
Blood counts						
Leukocytes [G/L]	172 (0%)	8.37 [5.62–12.2]	6.98 [2.88–10.6]	9.72 [6.04–12.9]	.**003**	0.808
Neutrophils [G/L]	172 (0%)	6.70 [4.25–10.5]	5.00 [2.20–9.10]	8.10 [4.90–11.4]	.**005**	0.958
Lymphocytes [G/L]	172 (0%)	0.7 [0.4–1.2]	0.7 [0.4–1.1]	0.7 [0.5–1.2]	.444	0.158
Thrombocytes [G/L]	172 (0%)	177 [113–239]	153 [110–220]	181 [128–245]	.136	0.651
Hemoglobin [mg/dL]	172 (0%)	12.6 [10.0–14.7]	11.8 [9.6–14.7]	13.0 [10.6–14.7]	.294	0.328
Specific Medication						
Oseltamivir	172 (0%)	168 (98%)	61 (100%)	107 (96%)	.134	0.992
Outcomes						
IAPA						
probable IAPA	172 (0%)	24 (14%)	4 (7%)	20 (18%)	.038	…
Deceased at data cut off	172 (0%)	107 (62%)	36 (60%)	71 (64%)	.522	…
Length of ICU stay	172 (0%)	7 [3–15]	7 [4–11]	8 [3–19]	.412	…

*P* denotes *P* values before ITPW weighting, *p*_IPTW_ denotes *P* values after IPTW adjustment. Bold values indicate statistical significance at *P* < .05.

BMI, body mass index; ICU, intensive care unit; COPD, chronic obstructive pulmonary disease; SOFA, sequential organ failure assessment; PEEP, positive end expiratory pressure; vvECMO, veno-venous extracorporeal membrane oxygenation, NIV, non-invasive ventilation, HFNC, high flow nasal cannula; IL-6, interleukin 6; CRP, C-reactive protein; IAPA, influenza-associated pulmonary aspergillosis. All parameters in Table 1 reflect values within the first 24 h after ICU admission. In total, 116 patients (68%) were intubated at some point during their ICU stay.

^a^Documented coronary heart disease either by specific coronary imaging or coronary angiography.

^b^Comprises immunosuppressive medication (low dose of glucocorticoids are excluded) as well as diseases with severe immunosuppression.

Among the 61 patients receiving early empirical mold-active antifungals, 57 (94%) were treated with standard-dose intravenous posaconazole, while three patients each (3%) received intravenous isavuconazole or voriconazole. Treatment continued until resolution of influenza-associated respiratory failure. All patients underwent therapeutic drug monitoring at least twice weekly, with adequate plasma concentrations confirmed in all cases. Serum and bronchoalveolar lavage (BAL) galactomannan tests were performed at similar frequencies in both groups 2 [1–3] serum GM tests and 1 [0–2] (*P* = .342) BAL GM tests per patient thereby minimizing potential observation bias (Supplementary Table 1). No patients receiving empirical antifungals required discontinuation due to adverse effects.

### Propensity Score

As shown in Table 1, the empirical antifungal treatment and control groups differed in baseline characteristics indicating potential non-random assignment bias. We used a 6-variable multivariable logistic regression model (Supplementary Table 2) to predict the propensity score (Supplementary Figure 5). After re-weighting, all between-group differences were eliminated (Table 1; Supplementary Figure 6). Additionally, the trimmed IPTW demonstrated similar effectiveness in reducing group differences, confirming the robustness of the propensity score (data not shown).

### IAPA Incidence and Early Empirical Administration of Antifungals

Within 30 days of ICU admission, 24 IAPA cases were observed, with a 1-, 15-, and 30-day incidence of 6.4% (95% CI: 3.6–11.3), 14.3% (9.5–20.8), and 15.2% (10.4–21.8), respectively (Supplementary Figure 7). No cases were diagnosed beyond 30 days. IAPA was identified after a median of 2 days (IQR: 0–7) post-admission, with diagnostic criteria including BAL galactomannan (GM) >1.0 ODI (23/24), BAL culture positive for *Aspergillus* species (20/24, all *Aspergillus fumigatus*), serum GM >0.5 ODI (14/24), and positive BAL *Aspergillus* PCR (17/24), alongside FUNDICU-defined radiologic criteria. Of the 17 patients who died after IAPA diagnosis, necropsy was performed on 14, with invasive aspergillosis confirmed histopathologically in 12 cases (n = 3 empirical antifungal treatment group and n = 9 without early empirical mold-active antifungal administration).

**Figure 1.**
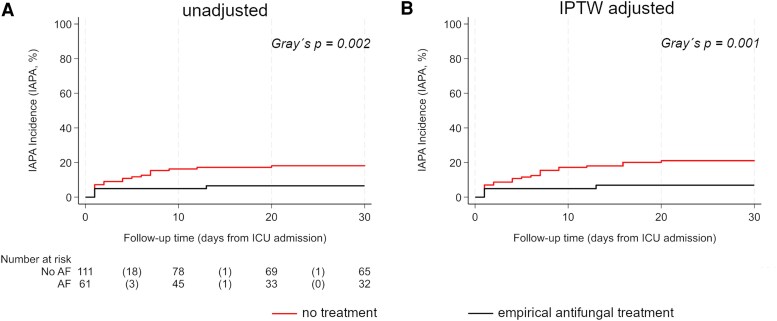
A graph showing 30-d IAPA incidence in patients with and without empirical antifungal treatment. A competing risk analysis was performed, considering death as a competing risk for IAPA development. Panel *A* represents the unadjusted analysis, while Panel *B* shows the IPTW-weighted (adjusted) analysis. AF, antifungal treatment; IAPA, influenza-associated pulmonary aspergillosis; ICU, intensive care unit.

Of the 24 IAPA cases, 4 were detected in patients with early empirical mold-active antifungal administration, with 2 cases detected at the day of ICU admission, one on the next day and one on day 13 after ICU admission. The latter case was classified as a breakthrough infection, as the serum posaconazole level remained above 1.4 mg/L (≥0.7 mg/L) 2 days before IAPA detection. The remaining 20 IAPA cases occurred in patients without empirical administration of antifungals (Supplementary Table 3), with 8 of 20 cases detected by tests performed within 48 h after ICU admission. In the unadjusted competing risk analysis, the 30-day IAPA incidence was 19.6% (95% CI: 13.1–28.8) in the non-empirical antifungal group and 7.1% (95% CI: 2.7–18.2) in the early empirical antifungals group (Gray's test, *P* = .002, Figure 1*A*). The Fine and Gray's model yielded a sub-distributional hazard ratio (SHR) for IAPA of 0.34 (95% CI: 0.12–0.98, *P* = .048) for early empirical administration of antifungals (within 24 h after ICU admission).

After re-weighting the data with the generated IPTW, the adjusted 30-day IAPA incidence was 20.4% in the group without early empirical administration of antifungals and 7.7% in the group with empirical antifungal treatment (Gray's test, *P* = .001, Figure 1*B*). The Fine and Gray's model with IPTW weighting showed an SHR of 0.21 (95% CI: 0.10–0.92, *P* = .045) for early empirical antifungal treatment (within 24 h after ICU admission).

In the group of patients without early empirical mold-active antifungal administration, the patients ultimately received antifungal treatment after diagnosis of IAPA (voriconazole in 13 patients, isavuconazole in 4, posaconazole in 2, and liposomal amphotericin B in 1 patient, respectively). In the group of patients receiving empirical antifungals early (ie within 24 hours after ICU admission and prior to IAPA diagnosis), 2 patients remained on posaconazole after diagnosis of IAPA, while one was switched to isavuconazole and another to voriconazole for the treatment of IAPA. Detailed patient characteristics are available in Supplementary Table 3.

### Empirical Antifungal Treatment and Survival

**Figure 2.**
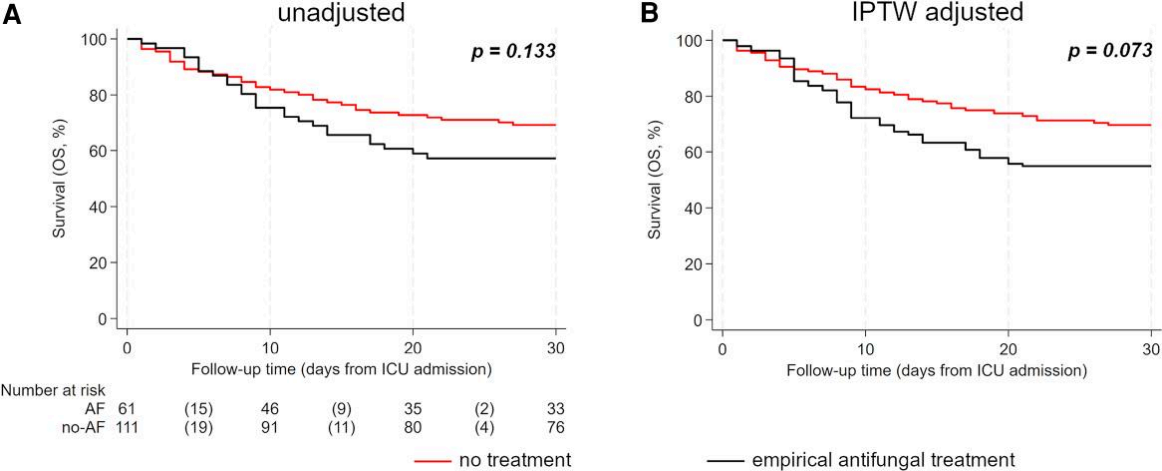
Thirty-day ICU survival based on empirical antifungal treatment. Panel *A* shows the unadjusted analysis, while Panel *B* presents the IPTW-adjusted analysis. *P*-values were calculated using the log-rank test. A risk table was included only for the unadjusted analysis. ICU survival was estimated using Kaplan–Meier curves. AF, antifungal treatment; IAPA, influenza-associated pulmonary aspergillosis.

Within 30 days of ICU admission, 60 deaths were recorded, yielding 1-, 15-, and 30-day ICU survival rates of 97.1% (95% CI: 93.1–98.7), 72.5% (65.2–78.6), and 64.8% (36.5–71.5) for the entire cohort (Supplementary Figure 6). In the time-to-event analysis, 30-day ICU survival was 57.2% (43.8–68.5) for patients receiving empirical mold-active antifungals, compared with 69.1% (59.6–76.7) in those not receiving it (*P =* .133) (Figure 2*A*). After re-weighting for IPTW to adjust for non-random assignment bias, the analysis still showed no significant difference in 30-day ICU survival between the two groups (log-rank *P* = .073) (Figure 2*B*). To investigate the potential effect of early empirical administration of antifungals on long-term ICU survival, we repeated the time-to-event analysis for 90-day ICU survival. Notably, no difference was observed between the two groups for this endpoint (Supplementary Figure 8).

**Figure 3.**
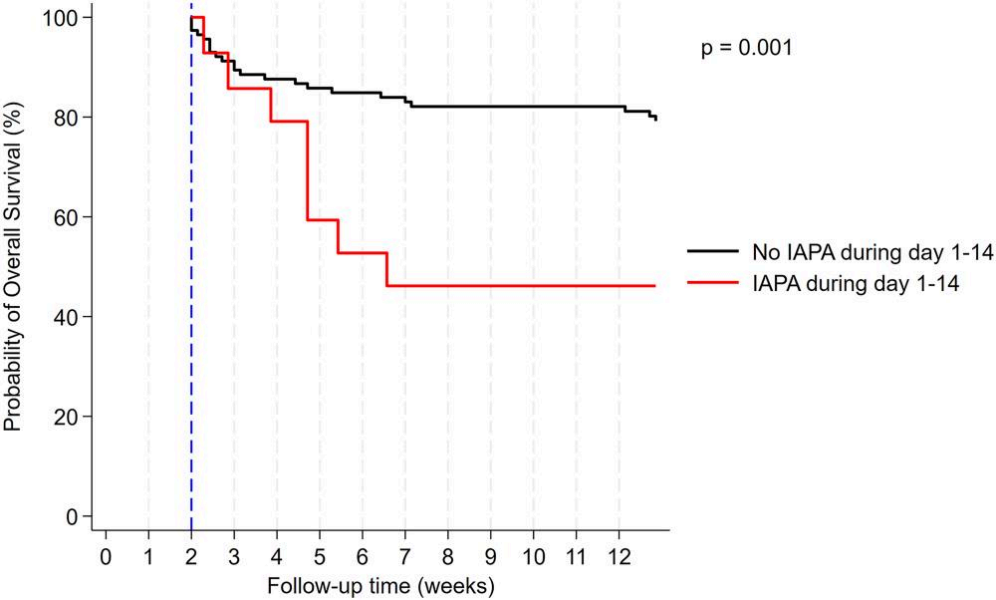
Landmark analysis of 90-d ICU survival based on IAPA diagnosis. Day 14 after ICU admission was chosen as the landmark date, as most IAPA diagnoses occurred prior to this time point. IAPA, influenza-associated pulmonary aspergillosis; ICU, intensive care unit.

Using univariable time-to-event regression, we assessed the impact of IAPA on 90-day ICU survival as another outcome. Patients who developed IAPA had significantly worse 90-day ICU mortality (HR: 2.13, 95% CI: 1.14–3.95, *P* = .017). These findings remained significant in multivariable regression adjusting for age, sequential organ failure assessment (SOFA), creatinine, comorbidities, body mass index (BMI), and PaO₂/FiO₂ (Supplementary Table 4). In a 14-day landmark analysis, 90-day ICU survival was 79.2% in non-IAPA patients vs 46.2% in IAPA patients (Mantel–Byar *P =* .001) (Figure 3).

### Benefits of Early Empirical Administration of Mold Active Antifungals in Patients With Influenza and Severe Respiratory Failure

We next aimed to identify patient groups that benefit most from this intervention. Univariable analysis of 30-day IAPA identified chronic obstructive pulmonary disease (COPD), prior malignancy, disease severity markers (eg, SOFA, lower PaO₂/FiO₂, higher PEEP), and high inflammatory markers (eg, C-reactive protein, ferritin, interleukin-6) as associated factors (Supplementary Table 5).

A subsequent multivariable analysis included all univariable predictors, selecting only the strongest when parameters were nested (PaO₂/FiO₂ for disease severity, CRP for inflammation). This identified PaO₂/FiO₂ and CRP as independent predictors of IAPA (Supplementary Table 6).

**Figure 4.**
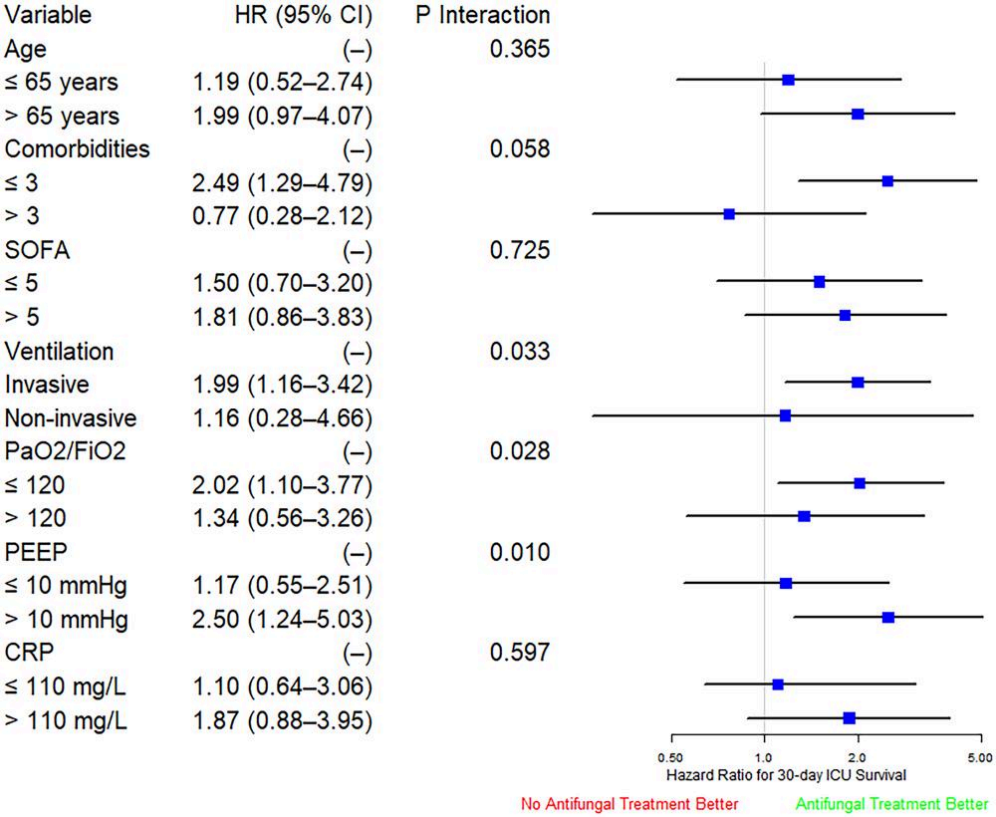
Subgroup analysis forest plot showing the relative association of empirical antifungal treatment with OS based on selected clinical co-variables. Squares represent the subgroup hazard ratios, with the bars indicating the 95% CIs. The vertical line marks the “line of unity,” where patients with and without empirical antifungal treatment have similar hazards of OS. Regression results were derived by fitting an interaction between preemptive antifungal treatment and the respective subgroup variable.

We then conducted subgroup analysis using IPTW-adjusted Cox models to examine the association between early empirical administration of antifungals and 30-day ICU survival. Our findings revealed an interaction between early empirical antifungals and respiratory parameters, particularly ventilation, PEEP, and PaO₂/FiO₂. This led us to explore the potential modifying effect of invasive ventilation on treatment outcomes. In this hypothesis-generating, post-hoc analysis, the beneficial effect of early empirical administration of antifungals appeared to be confined to patients with very severe respiratory failure (ie PaO₂/FiO₂ ≤120) (Figure 4).

## DISCUSSION

We conducted a multicenter study on 172 patients with influenza-related ARDS patients to assess early empirical antifungal treatment's impact on IAPA and survival. The cohort reflects typical critically ill patients, enhancing the study's generalizability [17].

The overall IAPA incidence in the cohort was 14% (24/172), consistent with previous studies. Additionally, 46% (11/24) of cases were diagnosed within the first 24 h after ICU admission, aligning with prior reports [7]. Given the high proportion of early IAPA cases shortly after ICU admission, we conclude that prophylactic antifungal administration cannot prevent these cases. However, antifungal prophylaxis based on risk profiles may be effective for later ICU periods. With IAPA incidence rates exceeding 20% in some critically ill influenza cohorts and early onset in half of the cases, empirical or preemptive treatment is necessary alongside prophylaxis, rather than relying on prophylaxis alone [11]. Based on local epidemiology, we propose incorporating empirical or preemptive antifungal therapy into an influenza care bundle for ICUs to address IAPA early. At our treatment centers, we implemented a guideline for empirical antifungal administration in critically ill patients with influenza and ARDS, though the decision was left to the treating physician due to limited outcome data at the time. This approach enabled the creation of case and control groups. We found that empirical antifungal treatment was associated with reduced IAPA rates. Empirical antifungal treatment was associated with a reduction of IAPA incidence by 79% (20% to 8%) at 30 days, mainly by preventing late-onset cases. Only one breakthrough infection occurred in the treatment group [18]. Our findings align with the POSAFLU study, which showed a reduction in incidence from 11% to 5%, though without statistical significance. The POSAFLU study was limited by being underpowered and restricting antifungal prophylaxis to 7 days, which potentially allowed fungal infections to develop afterwards in patients with persisting risk factors for IAPA. In our cohort, empirical administration of mold active antifungals within 24 h after ICU admission could retrospectively be considered as preemptive or targeted IAPA treatment in those patients diagnosed early with IAPA and antifungal prophylaxis in the others. With antifungal prophylaxis, some of the 12 IAPA cases that occurred later during the course of ICU treatment in patients without antifungals, might have been preventable. However, mold-active antifungals may reduce GM test sensitivity, potentially impacting accurate IAPA diagnosis, especially in prophylactic use [19, 20]. In our cohort, three non-IAPA patients showed borderline BAL GM positivity and positive Aspergillus PCR. After empirical posaconazole treatment, GM levels dropped, and PCR tests turned negative (Supplementary Figure 9).

Although empirical antifungal treatment was associated with reduced IAPA incidence, it did not improve short- or long-term ICU survival, suggesting it may not be optimal for all critically ill influenza patients. Currently, no clear guidelines exist for the early empirical use of antifungals in ICU patients with viral-induced pneumonia [21]. To refine patient selection, we conducted a subgroup interaction analysis, which indicated a potential benefit of empirical antifungal treatment in patients with severe respiratory failure (PaO₂/FiO₂ ≤120). This finding serves as a hypothesis-generating basis for future randomized trials, particularly in this high-risk subgroup, to further investigate the efficacy of this approach. This approach can support antifungal stewardship by limiting indiscriminate use and reducing resistance risk [22].

Choosing the best antifungal agent for empirical administration of antifungals is another important issue [23]. Previous reports indicate that antifungals exhibit varying pharmacokinetic properties in ICU patients due to larger volumes of distribution and the use of extracorporeal circuits [24–26]. In our study, intravenous posaconazole was the most commonly used antifungal. Its efficacy was monitored through therapeutic drug monitoring in all patients, ensuring sufficient plasma concentrations and excluding therapeutic failure.

Our study has limitations, including non-randomized antifungal use, ICU variability between groups, and possible underreporting of adverse events. Mortality remained higher in the treatment group despite lower IAPA rates, possibly due to unmeasured confounders or IAPA reflecting disease severity rather than causing death. However, we observed comparable baseline characteristics between patients with and without early empirical antifungal administration, and we subsequently adjusted for any imbalances using propensity score and IPTW-weighted analysis. Although we conducted a multicenter study, the centers are located within a limited geographic area, and all *Aspergillus* isolates were azole-sensitive. This may limit the generalizability of our findings to regions with lower overall incidence of IAPA or high rates of azole-resistant *Aspergillus* strains [27]. Given the emergence of novel antifungals and growing awareness of dosing challenges in the ICU setting, these considerations should be incorporated into future trials and treatment strategies [28].

It is important to note that in our cohort, non-intubated patients underwent sputum culture and GM testing as a surrogate for BAL-based diagnostics, a standard approach in our clinical setting. None of these tests yielded positive results. Although recent evidence supports the feasibility of this strategy, the FUNDICU algorithm does not accommodate non-BAL samples, limiting classification. Thus, we believe the reduced sensitivity in these cases reflects a limitation of the classification system rather than the diagnostic strategy itself [8, 29]. We confirmed the high incidence of IAPA in critically ill influenza patients, with more than half of cases diagnosed within the first 48 h of ICU admission, making these early cases unpreventable by prophylaxis. Additionally, we observed a poor survival rate of only 46% in IAPA patients, as shown by a multi-state post-event model with landmark analysis. This highlights the need for future research to identify patients who may benefit from early empirical antifungal treatment or later prophylaxis as part of an ICU influenza care bundle. Our results suggest that the other half of IAPA cases (late onset) could potentially be prevented with prophylaxis. In our hypothesis-generating-analysis, patients with severe respiratory failure appeared to potentially benefit the most from this strategy, though further research is needed to confirm this observation. Our results might be able to guide further studies in this field and should be independently validated, ideally through a prospective randomized trial.

## Supplementary Material

ciaf507_Supplementary_Data

## Supplementary Data

Supplementary materials are available at *Clinical Infectious Diseases* online. Consisting of data provided by the authors to benefit the reader, the posted materials are not copyedited and are the sole responsibility of the authors, so questions or comments should be addressed to the corresponding author.
