# Supplementary material for: Empirical Antifungal Treatment of Critically Ⅲ Patients With Influenza-Associated Acute Respiratory Distress Syndrome: A Propensity Score Weighted Observational Study
Source: Clin Infect Dis. 2025 Sep 15;82(3):e485–93. doi: 10.1093/cid/ciaf507 (PMC13016648; doi:10.1093/cid/ciaf507)
Supplement: ciaf507_Supplementary_Data [file ciaf507_supplementary_data.docx]

**Supplementary Methods:**

Propensity score matching:

The propensity score (*e*) was derived from a multivariable logistic regression model, with early empirical antifungal administration as the outcome variable. To ensure at least three events per predictor variable, we limited the model to a maximum of 10 predictors. Variables were selected if they had a *p*-value ≤ 0.15 and/or an SMD ≥ 0.30.

The propensity score (*e*) was then transformed into an inverse-probability-of-treatment-weight (*IPTW*) using the average treatment effect principle:

*IPTW* $=\frac{( empirical antifungal treatment)}{e}+\frac{1-(empirical antifungal treatment)}{1-e}$ where administration of an antifungal represents the treatment assignment. [1, 2] For balance diagnostics, we then re-estimated SMDs and p-values for difference between the two groups with the IPTW-weighted data. For this propensity score model, missing data of three variables (lactate, interleukin 6, ferritin) were imputed using a chained equations algorithm

Sensitivity analyses included a trimmed IPTW (i.e. excluding patients with an IPTW ≤ the 1^st^ and ≥ the 99^th^ percentile of its distribution). [3]

Associations between empiric antifungal treatment and time-to-event outcomes were evaluated using univariable unweighted and inverse probability of treatment weight (IPTW)-adjusted Cox models, as well as Fine and Gray sub-distribution hazard models. Risks of IAPA development and all-cause mortality were assessed using competing risk cumulative incidence estimators, Gray’s tests, and sub-distribution hazard models, treating death as a competing event in IAPA risk analyses. The proportional hazards assumption was assessed using Schoenfeld residuals for all survival models.

To address immortal time bias, IAPA occurrence was modeled as a time-dependent variable in multi-state Cox models. Its association with ICU survival was examined as a co-secondary outcome by splitting follow-up into pre- and post-IAPA periods. A 14-day landmark analysis was conducted to visualize ICU survival, as most IAPA cases were diagnosed within the first two weeks of ICU admission.

In an exploratory, hypothesis-generating analysis, interactions between treatment and pre-specified subgroups (age ≤/> 65 years, comorbidities ≤/> 3, sequential organ failure assessment (SOFA) ≤/> 5, P_a_O2/FiO_2_ ≤/> 120, invasive vs. non-invasive ventilation, positive-end-expiratory pressure (PEEP) ≤/> 10, and c-reactive protein (CRP) ≤/> 110 mg/L) were assessed to explore potential effect modification of early empiric antifungal treatment on 30-day ICU survival. Subgroup cutoffs were chosen based on variable medians.

The full dataset and main analysis code are available upon request from the first author (SH). A p-value < 0.05 was considered statistically significant in all analyses.

**References**

1. Chesnaye NC, Stel VS, Tripepi G, et al. An introduction to inverse probability of treatment weighting in observational research. Clin Kidney J, **2021**; 15: 14–20.

2. Austin PC, Stuart EA. Moving towards best practice when using inverse probability of treatment weighting (IPTW) using the propensity score to estimate causal treatment effects in observational studies. Stat Med, **2015**; 34: 3661–79.

3. Stürmer T, Webster-Clark M, Lund JL, et al. Propensity score weighting and trimming strategies for reducing variance and bias of treatment effect estimates: A simulation study. Am J Epidemiol, **2021**; 190: 1659–70.

4. Segal BH, Herbrecht R, Stevens DA, et al. Defining responses to therapy and study outcomes in clinical trials of invasive fungal diseases: Mycoses study group and european organization for research and treatment of cancer consensus criteria. Clin Infect Dis, **2008**; 47: 674–83.


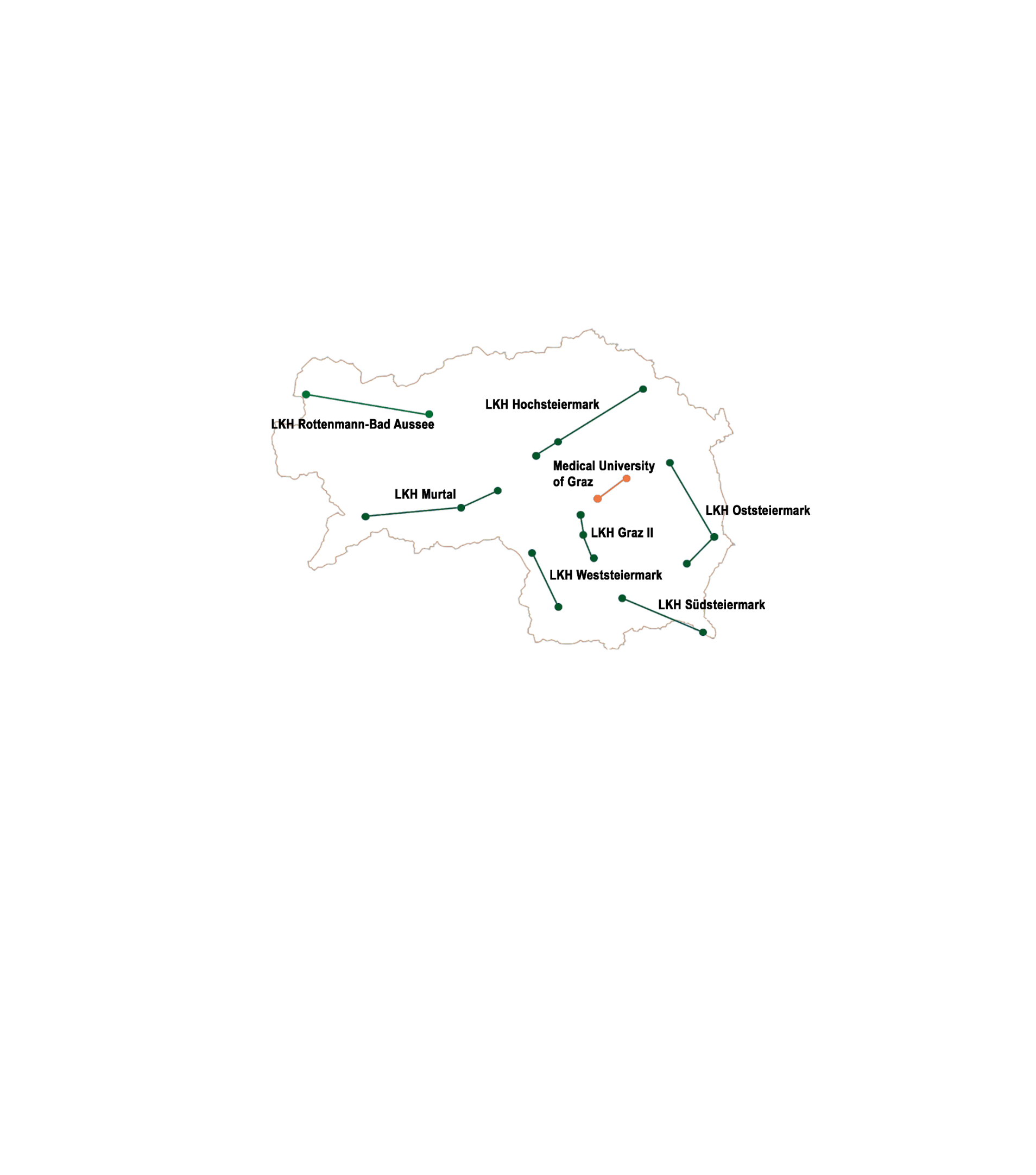


| **Center** | **n** |
| --- | --- |
| LKH-Graz (Internal Medicine) | 106 |
| LKH-Graz (Surgery) | 36 |
| LKH-Graz (Neurology) | 3 |
| LKH-Oststeiermark (Mixed) | 5 |
| LKH-Südsteiermark (Mixed) | 3 |
| LKH-Weststeiermark (Mixed) | 3 |
| LKH-Hochsteiermark (Mixed) | 5 |
| LKH-Graz II (Mixed) | 9 |
| LKH-Rottenmann/ Bad Aussee (Mixed) | 2 |

**Supplementary Figure 1: Treatment centers involved in the study** Each point in the figure represents a participating hospital, with connecting lines indicating the hospital network structure (LKH – hospital network). *n* = number of patients treated at each center. Note that patients are displayed according to the hospital where they received treatment, not necessarily their initial ICU admission site. To ensure comprehensive inclusion, patients from non-ECMO centers with comparable severity were also enrolled, even if not transferred to ECMO-capable facilities. The ECMO centers were the Medical University of Graz and LKH Graz II.


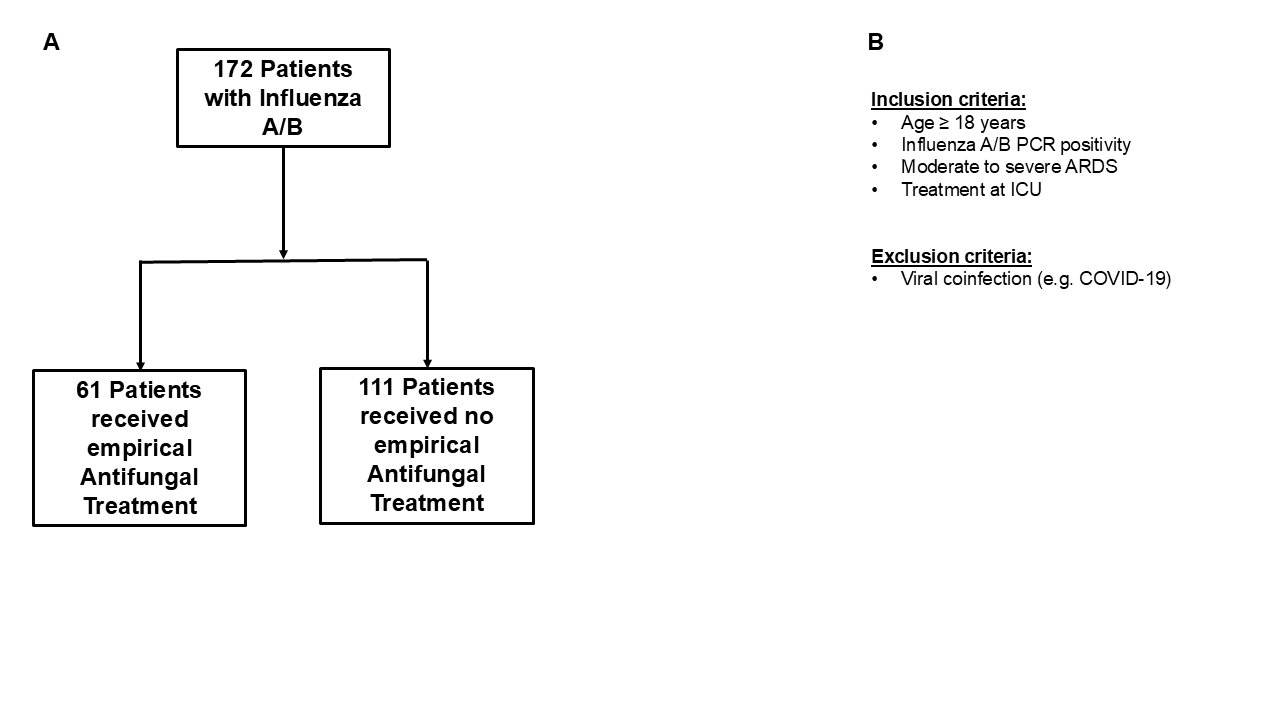


**Supplementary Figure 2:** Full trial protocol and flow diagram. **A)** 172 patients were included in the analyses, whereof 61 received empirical antifungal treatment and 111 did not. **B)** Inclusion and exclusion criteria of the study. COVID-19 = coronavirus disease 2019; PCR = polymerase chain reaction; ICU = intensive care unit


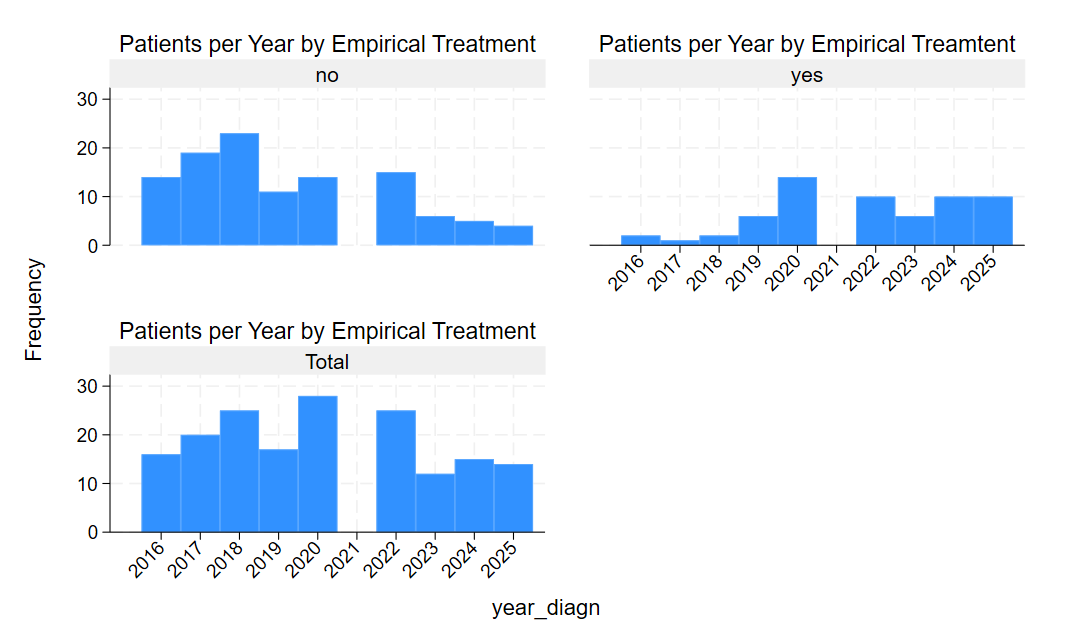


**Supplementary Figure 3:** Histogram showing the annual case load of influenza patients. The upper left panel displays patients without empirical antifungal treatment, the upper right panel shows those who received empirical antifungal treatment, and the lower left panel depicts the total number of influenza patients admitted to our ICUs.


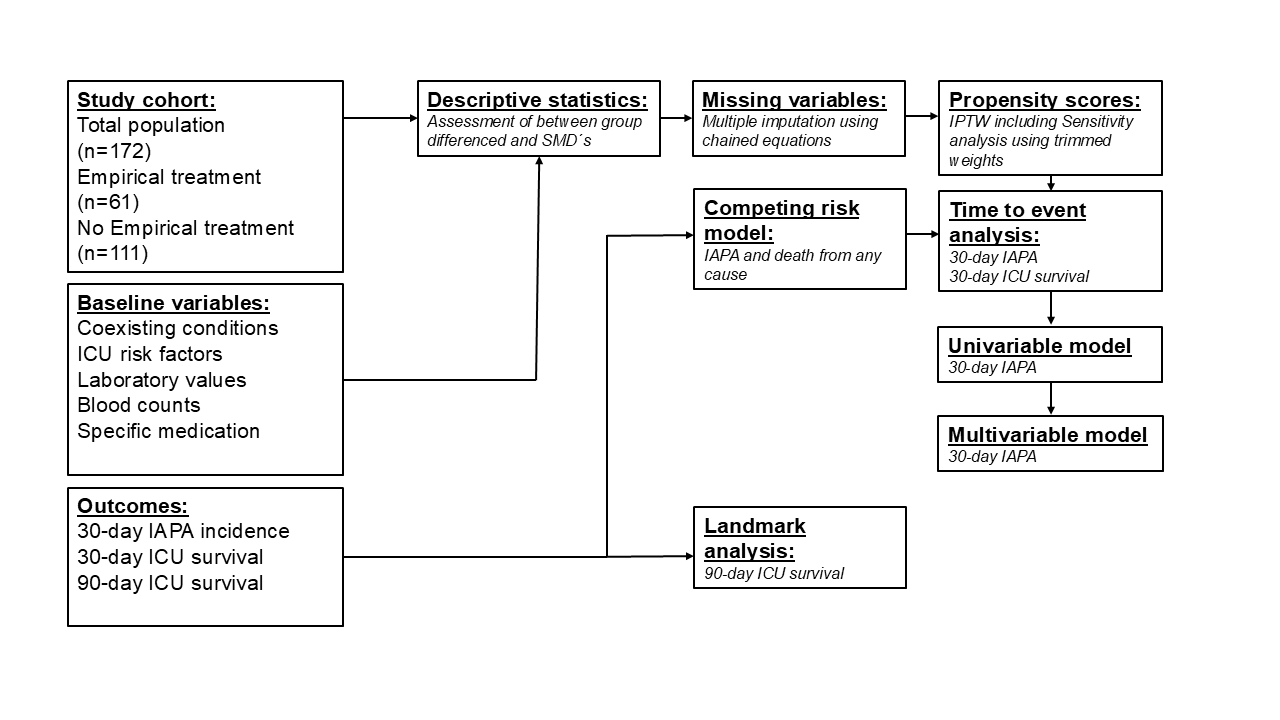

**Supplementary Figure 4:** Statistical analysis plan.

IAPA- influenza associated pulmonary aspergillosis; ICU- intensive care unit; SMD – standardized mean difference; IPTW – invers probability of treatment weight

**A**

**B**


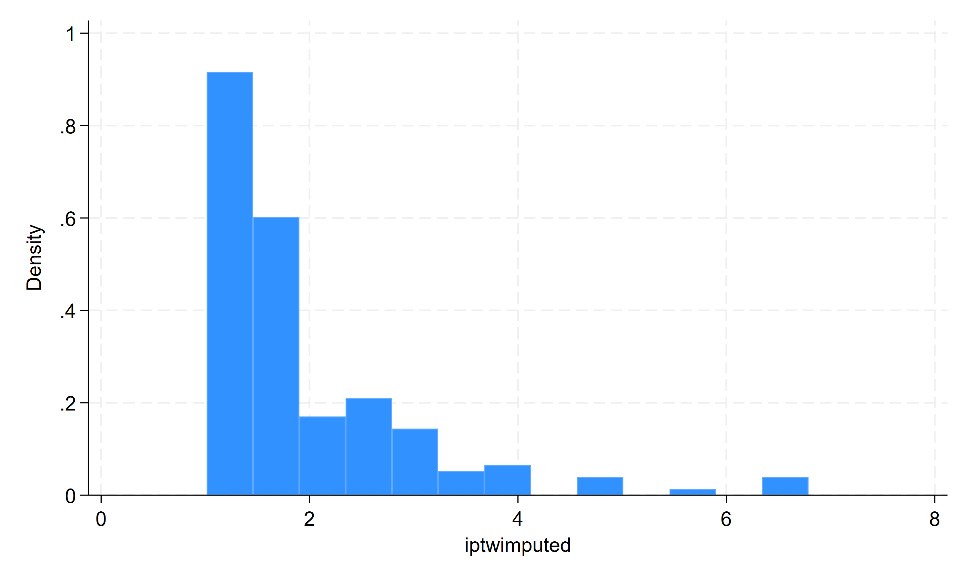

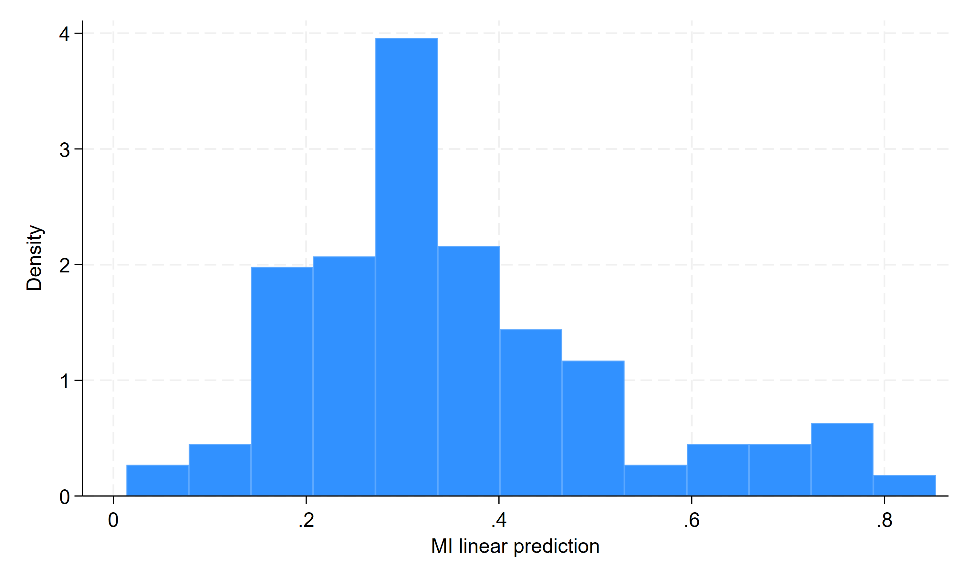


**Supplementary Figure 5:** Histograms of the Propensity Score and the IPTW. (A) The propensity score can range from 0 to 1. Multiply by 100 to obtain probabilities (in percent) of having received mold active empirical antifungal treatment. (B) The IPTW was defined as the inverse of the probability of receiving the treatment that the patient received.


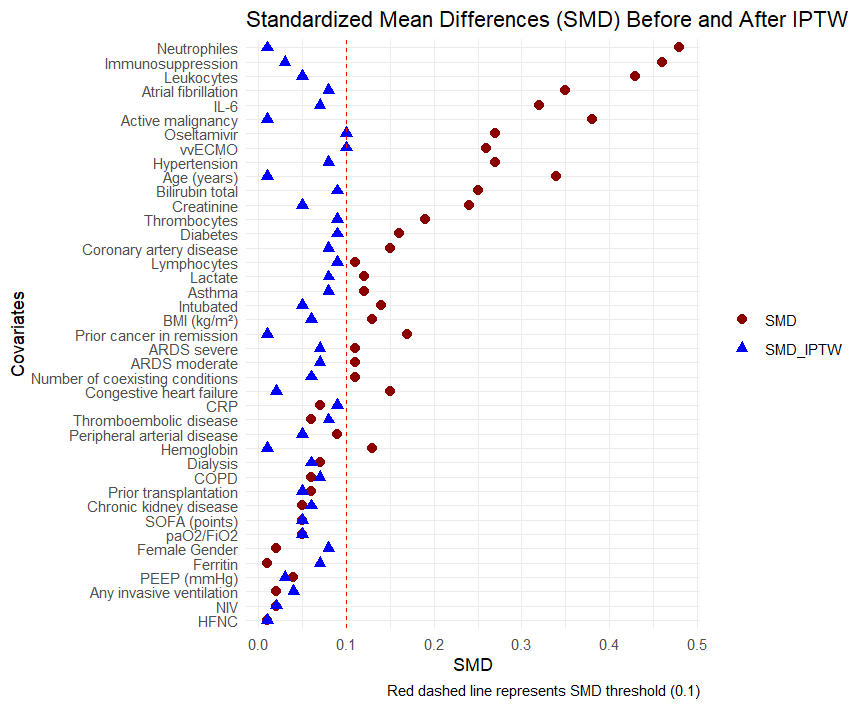


**Supplementary Figure 6:** Standardized mean difference (SMD) plot. Red dots denote Standardized mean differences (SMDs) before weighting the raw inverse of the probability of treatment weight (IPTW). Blue triangels denote the SMDs after weighting the IPTW. The constructed propensity score was able to balance all variables below SMD of 0.1 resulting no significant differences in between the treatment groups.

HFNC – high flow nasal cannula, NIV – noninvasive ventilation; SOFA – sequential organ failure assessment; COPD – chronic obstructive pulmonary disease, PEEP – positive end-expiratory pressure; BMI – body mass index; ECMO – extra corporeal membrane oxygenation; IL-6 – interleukin-6; CRP – C reactive protein


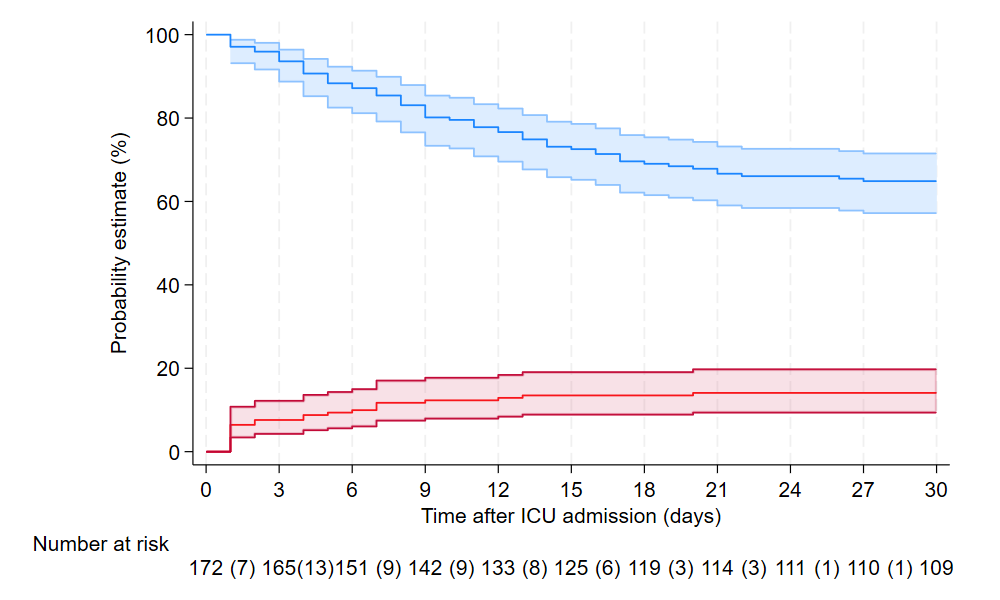


**Supplementary Figure 7:** Overall survival and IAPA incidence displayed as competing events of the whole cohort (n=172) 30-day survival estimates (64.8% [57.2-71.5], blue curve) and IAPA incidence estimate (15.2% [10.4-21.8], red curve) of the total cohort. Abbreviation: ICU = intensive care unit.

**A B**

**
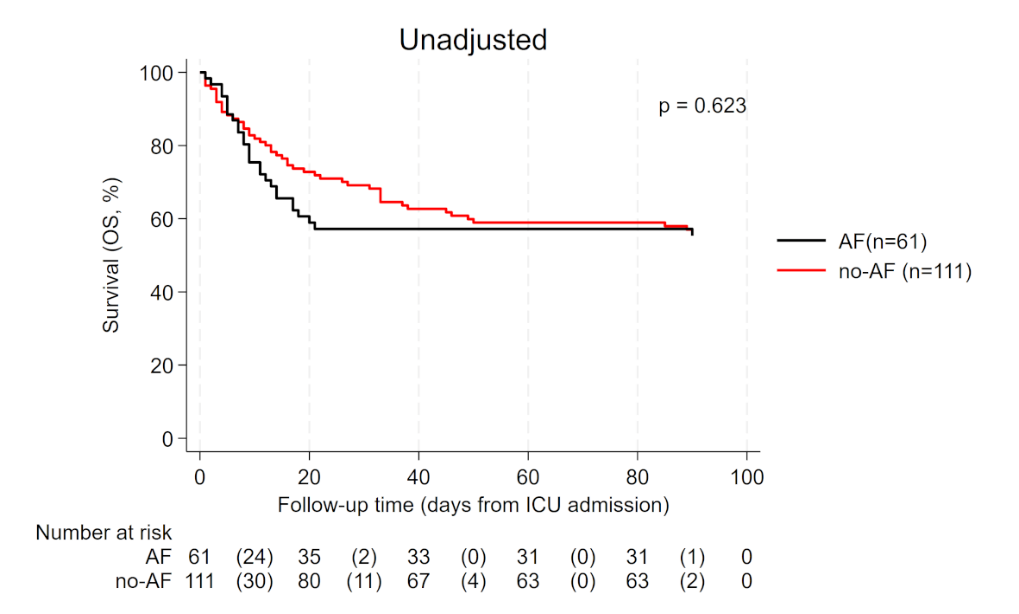

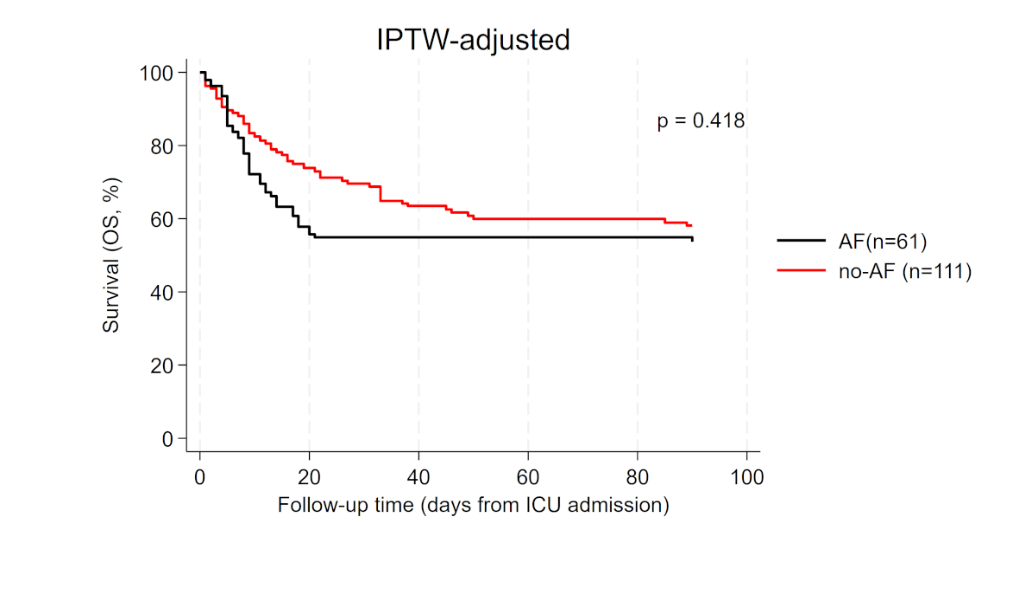
**

**Supplementary Figure 8: 90-day ICU survival according to empirical antifungal treatment (Long-term survival)**

A) Unadjusted analysis B) IPTW adjusted analysis. p values are calculated using the log rank test. Risk table was only computed for the unadjusted analysis. ICU survival was calculated by using Kaplan Maier estimators. IAPA – Influenza associated pulmonary aspergillosis; AF – empirical antifungal treatment.


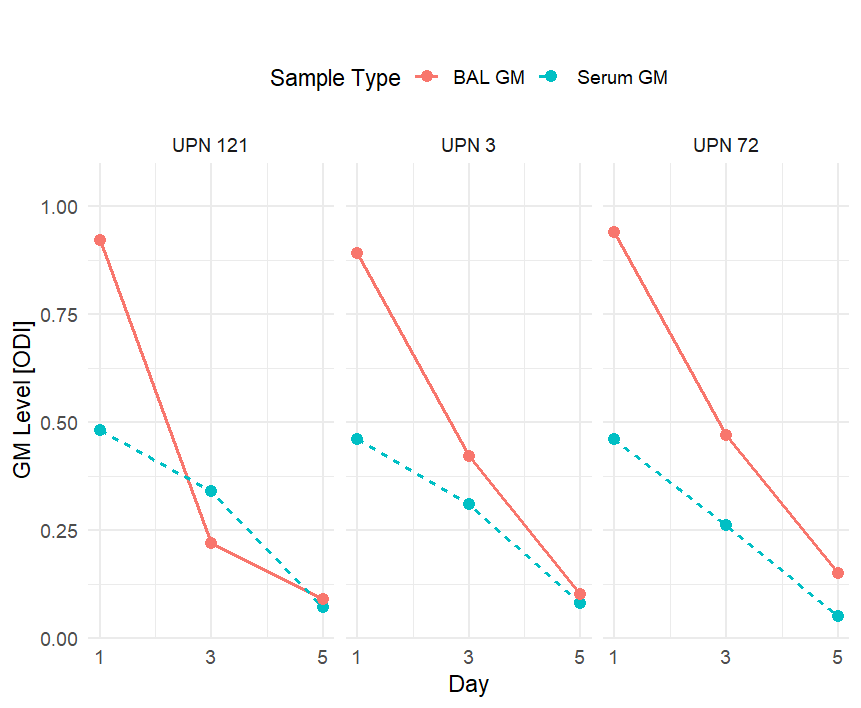


**Supplementary Figure 9: Trends of Serum and BAL Galactomannan (GM) levels of three patients with borderline positive GM-tests receiving empirical posaconazole treatment.** GM levels in serum and bronchoalveolar lavage fluid (BAL) were measured on Days 1, 3, and 5 following ICU admission. Solid lines represent serum GM values, and dashed lines represent BAL GM values. Units are reported as optical density index (ODI). Aspergillus PCR results are annotated for each patient, showing positive results on Day 1 and negative results by Day 5. The decline in GM levels and conversion to PCR negativity suggest a treatment response to early empirical antifungal therapy.

| **Variable** |  | **n (%miss)** | **Overall**  **(n=172)** | **Early Empirical Antifungal Treatment**  **(n=61)** | **No Early Empirical Antifungal Treatment**  **(n=111)** | ***p*** |
| --- | --- | --- | --- | --- | --- | --- |
|  |  |  |  |  |  |  |
| ICU weeks |  | 172 (0%) | 1.7 [0.6-3.7] | 1.9 [0.9-3.3] | 1.7 [0.6-3.5] | 0.657 |
| Serum-GM per ICU week |  | 172 (0%) | 0.6 [0.0-1.2] | 0.6 [0.0-1.3] | 0.5 [0.0-1.6] | 0.823 |
| BAL-GM per ICU week |  | 172 (0%) | 0.0 [0.0-0.3] | 0.0 [0.0-0.3] | 0.0 [0.0-0.2] | 0.393 |
| BAL GM per patient (total ICU stay |  | 172 (0%) | 1,09  (188/172) | 1,08  (66/61) | 1,09  (122/111) | 0.875 |
| BAL culture per patient (total ICU stay |  | 172 (0%) | 1,01  (174/172) | 0.96  (59/61) | 1,03  (115/111) | 0.324 |

**Supplementary Table 1**: Galactomannan testing per ICU week.

No differences in testing frequencies could be observed between the groups. P values were calculated via rank-sum tests. BAL GM per patient (total ICU stay) indicates the average number of BAL GM tests performed per patient during the ICU stay, including follow-up BALs, shown in parentheses as (total BAL GM tests / number of patients in the cohort). BAL culture per patient (total ICU stay) indicates the average number of BAL aspergillus tests performed per patient during the ICU stay, including follow-up BALs, shown in parentheses as (total BAL GM tests / number of patients in the cohort). GM = galactomannan BAL = broncho-alveolar-lavage, ICU = intensive care unit

| **Variable** |  | **Multivariable Odds Ratio (OR)** | **95%CI** | **P** |
| --- | --- | --- | --- | --- |
|  |  |  |  |  |
| **Demographic variables** |  |  |  |  |
| Age (per 1 year increase) |  | 0.97 | [0.93-0.56] | 0.294 |
| Active Malignant Disease |  | 0.56 | [0.04-7.47] | 0.661 |
| Immunosuppression |  | 2.16 | [0.14-34.45] | 0.585 |
| Interleukin-6 (per 1 unit increase) |  | 0.99 | [0.99-1.00] | 0.609 |
| Bilirubin (per 1 unit increase) |  | 0.73 | [0.22-2.41] | 0.035 |
| Abs. Neutrophile Count (per 1 unit increase) |  | 0.86 | [0.76-0.99] | 0.035 |

**Supplementary Table 2:** A propensity score model for treatment group assignment

The table shows the predicted 6-variable propensity score model.

| **Patient Identifier** | **Group** | **Mode of Ventilation/oxygen supply** | **paO_2_/FiO_2_** | **Days to first test indicating IAPA*** | **Type of first test indicating IAPA** | **Asp-culture from BAL** | **Treatment of IAPA** | **Treatment success**** | **Outcome** |
| --- | --- | --- | --- | --- | --- | --- | --- | --- | --- |
| UPN 33 | treatment | invasive | 92 | 13 | BAL-GM | Asp. fum. | Posaconazole | no | deceased |
| UPN 47 | treatment | invasive | 102 | 0 | BAL-GM | / | Posaconazole | no | deceased |
| UPN 171 | treatment | non-invasive | 189 | 0 | Serum-GM | Asp. fum. | Voriconazole | yes | alive |
| UPN 172 | treatment | invasive | 75 | 1 | BAL-culture | Asp. fum. | Isavuconazole | no | deceased |
| UPN 6 | no-treat | invasive | 80 | 20 | BAL-GM | Asp. fum. | Ampho B | no | deceased |
| UPN 23 | no-treat | ECMO | 38 | 2 | BAL-GM | Asp. fum. | Voriconazole | no | deceased |
| UPN 26 | no-treat | invasive | 159 | 7 | BAL-culture | Asp. fum. | Voriconazole | no | deceased |
| UPN 56 | no-treat | invasive | 63 | 5 | BAL-GM | Asp. fum. | Voriconazole | no | deceased |
| UPN 67 | no-treat | invasive | 86 | 9 | BAL-GM | / | Voriconazole | no | deceased |
| UPN 74 | no-treat | invasive | 92 | 0 | BAL-culture | Asp. fum. | Voriconazole | no | deceased |
| UPN 80 | no-treat | ECMO | 64 | 4 | BAL-GM | / | Voriconazole | no | deceased |
| UPN 85 | no-treat | invasive | 99 | 4 | BAL-GM | Asp. fum. | Isavuconazole | yes | alive |
| UPN 105 | no-treat | invasive | 95 | 1 | BAL-GM | / | Posaconazole | no | deceased |
| UPN 122 | no-treat | ECMO | 51 | 0 | BAL-GM | Asp. fum. | Isavuconazole | no | deceased |
| UPN 139 | no-treat | invasive | 68 | 0 | BAL-GM | Asp. fum. | Voriconazole | no | deceased |
| UPN 150 | no-treat | ECMO | 53 | 7 | BAL-culture | Asp. fum. | Voriconazole | yes | alive |
| UPN 157 | no-treat | ECMO | 74 | 1 | BAL-GM | Asp. fum. | Voriconazole | yes | alive |
| UPN 158 | no-treat | invasive | 79 | 2 | BAL-GM | Asp. fum. | Isavuconazole | yes | alive |
| UPN 159 | no-treat | invasive | 62 | 1 | BAL-GM | Asp. fum. | Voriconazole | yes | alive |
| UPN 160 | no-treat | non-invasive | 120 | 1 | Serum-GM | Asp. fum. | Posaconazole | no | deceased |
| UPN 161 | no-treat | ECMO | 59 | 7 | BAL-GM | Asp. fum. | Voriconazole | no | deceased |
| UPN 162 | no-treat | invasive | 69 | 12 | BAL-GM | Asp. fum. | Isavuconazole | no | deceased |
| UPN 163 | no-treat | invasive | 102 | 6 | BAL-culture | Asp. fum. | Voriconazole | yes | alive |
| UPN 164 | no-treat | invasive | 145 | 1 | BAL-culture | Asp. fum. | Voriconazole | no | deceased |

**Supplementary Table 3: Characteristics of the IAPA patients.**

UPN – unified patient number; treatment – empirical antifungal treatment; no-treat – no empirical antifungal treatment; IAPA – influenza associated pulmonary aspergillosis; GM – galactomannan; BAL – broncho alveolar lavage; Asp – aspergillus; fum – fumigatus; PCR – polymerase chain reaction; Outcome denotes the status of the patients at data cut off. *Days to first test for IAPA indicate the time frame until performance of the IAPA defining test. ** Treatment success was defined according to the 2008 definition of the Mycoses Study Group and European Organization for Research and Treatment of Cancer Consensus Criteria [4]

| **Variable** | **90-day ICU survival** |
| --- | --- |
|  | **HR (95%CI, p)** |
|  |  |
| IAPA | 2.13 (1.14-3.95, **p=0.017**) |
| Age per year | 1.01 (0.99-1.02, p=0.326) |
| SOFA per point | 1.17 (1.03-1.33, **p=0.010**) |
| Creatinine per mg/dl | 0.92 (0.79-1.07, p=0.301) |
| Number of conditions | 1.16 (1.03-1.31, **p=0.009**) |
| BMI per kg/m² | 0.98 (0.94-1.02, p=0.498) |
| paO_2_/FiO_2_ | 0.99 (0.94-1.00, p=0.074) |

**Supplementary Table 4:** A multivariable cox regression model for 90-day ICU survival for adjustment of post-event IPAP for 6 important predictors of ICU survival

IAPA – influenza associated pulmonary aspergillosis; SOFA-sequential organ failure assessment; BMI – body mass index

| **Variable** |  | **Univariable Hazard Ratio** | **95%CI** | **P** |
| --- | --- | --- | --- | --- |
| Empirical antifungal treatment |  | 0.35 | 0.12-0.98 | **0.047** |
| **Demographic variables** |  |  |  |  |
| Age (per 5 years increase) |  | 0.97 | 0.88-1.07 | 0.596 |
| Female gender |  | 0.66 | 0.28-1.61 | 0.370 |
| BMI (per 5 kg/m² increase) |  | 1.25 | 0.98-1.61 | 0.075 |
| **Coexisting conditions** |  |  |  |  |
| Hypertension |  | 1.67 | 0.73-3.81 | 0.224 |
| Diabetes |  | 1.64 | 0.70-3.85 | 0.250 |
| Atrial fibrillation |  | 1.23 | 0.49-3.10 | 0.660 |
| Coronary heart disease |  | 1.60 | 0.68-3.74 | 0.278 |
| Congestive heart failure |  | 0.95 | 0.39-2.29 | 0.911 |
| Peripheral arterial disease |  | 1.83 | 0.43-7.81 | 0.411 |
| Thromboembolic disease |  | 0.72 | 0.17-3.08 | 0.664 |
| Chronic kidney disease |  | 0.90 | 0.31-2.63 | 0.849 |
| Dialysis |  | 0.59 | 0.08-4.39 | 0.610 |
| COPD |  | 0.11 | 0.01-0.80 | **0.030** |
| Prior cancer |  | 4.95 | 1.47-16.67 | **0.010** |
| Active cancer |  | 0.29 | 0.04-2.17 | 0.230 |
| Prior transplantation (SOT, stem-cell) |  | 5.32 | 1.81-15.63 | 0.002 |
| Immunosuppression |  | 1.55 | 0.58-4.17 | 0.379 |
| **ICU risk stratification** |  |  |  |  |
| SOFA (per 1 point increase) |  | 1.22 | 1.03-1.44 | **0.021** |
| paO_2_/FiO_2_ (per 50 units increase) |  | 0.33 | 0.18-0.61 | **<0.001** |
| PEEP (per 1 cmH_2_O increase) |  | 1.21 | 1.11-1.33 | <**0.001** |
| Severe ARDS (Global definition) |  | 4.35 | 1.72-10.97 | **0.002** |
| Any invasive ventilation |  | 11.13 | 2.61-47.49 | **0.001** |
| **Laboratory values** |  |  |  |  |
| Lactate (per 1 mmol/l increase) |  | 0.93 | 0.74-1.17 | 0.589 |
| IL-6 (per 500 pg/ml increase) |  | 1.03 | 1.01-1.05 | **0.005** |
| CRP (per 50 mg/l increase) |  | 1.26 | 1.11-1.42 | <**0.001** |
| Ferritin (per 1000 ng/ml increase) |  | 1.28 | 1.14-1.43 | <**0.001** |
| Creatinine (per 1mg/dl increase) |  | 1.12 | 0.95-1.34 | 0.178 |
| Bilirubin (per 1mg/dl increase) |  | 1.07 | 0.69-1.66 | 0.741 |
| **Blood counts** |  |  |  |  |
| Leukocytes [per 1 G/l increase] |  | 1.02 | 0.95-1.09 | 0.504 |
| Neutrophils [per 1 G/l increase] |  | 1.03 | 0.97-1.12 | 0.314 |
| Lymphocytes [per 0.5 G/l decrease] |  | 0.69 | 0.45-1.05 | 0.085 |
| Thrombocytes [per 100 G/l increase] |  | 0.69 | 0.42-1.14 | 0.154 |

**Supplementary Table 5:** Univariable Predictors of IAPA.

CI – confidence interval; BMI – body mass index; SOFA – sequential organ failure assessment; paO2/FiO2 - ratio of partial pressure of oxygen in blood (PaO2 and the fraction of oxygen in the inhaled air (FiO2); COPD – chronic obstructive pulmonary disease; SOT – solid organ transplant; PEEP – positive end-expiratory pressure; ARDS – acute respiratory distress syndrome; SOFA – sequential organ failure assessment; IL-6 interleukin-6; CRP C-reactive protein;

| **n** | **Variable** | **Multivariable Hazard Ratio** | **95%CI** | **p** |
| --- | --- | --- | --- | --- |
|  |  |  |  |  |
| 172 | Empirical antifungal treatment | 0.45 | 0.14-1.35 | 0.154 |
| 172 | Prior cancer | 3.14 | 0.81-12.17 | 0.094 |
| 172 | COPD | 0.28 | 0.03-2.27 | 0.236 |
| 172 | C-reactive protein (per 50 mg/l increase) | 1.15 | 1.00-1.32 | **0.041** |
| 172 | PaO_2_/FiO_2_ (per 50 units increase) | 0.47 | 0.25-0.89 | **0.022** |

**Supplementary Table 6:** Multivariable Cox regression of 30-day IAPA**.** n denotes the patients included in the model. COPD – chronic obstructive pulmonary disease;

**Alt text section:**

Supplementary Figure 1:

Treatment centers involved in the study. Each point represents a single hospital, with connecting lines indicating hospital networks. LKH represents the hospital network, and 'n' indicates the number of participants enrolled by each center.

Supplementary Figure 2:

Full trial protocol and flow diagram. A) A total of 172 patients were included in the analyses, with 61 receiving empirical antifungal treatment and 111 not. B) Inclusion and exclusion criteria for the study. Abbreviations: COVID-19 – coronavirus disease 2019, PCR – polymerase chain reaction, ICU – intensive care unit

Supplementary Figure 3:

Histogram showing the annual case load of influenza patients. The upper left panel displays patients without empirical antifungal treatment, the upper right panel shows those who received empirical antifungal treatment, and the lower left panel depicts the total number of influenza patients admitted to our ICUs

Supplementary Figure 4:

Statistical analysis plan. The schematic process illustrates how data were analyzed during the project, with missing variables accounted for using multiple imputations via chained equations. Abbreviations: IAPA – influenza-associated pulmonary aspergillosis, ICU – intensive care unit, SMD – standardized mean difference, IPTW – inverse probability of treatment weighting, SW – stabilized weights

Supplementary Figure 5:

Histograms of the Propensity Score and the IPTW. (A) The propensity score ranges from 0 to 1, and when multiplied by 100, it represents the probability (in percent) of receiving mold-active empirical antifungal treatment. (B) The IPTW is defined as the inverse of the probability of receiving the treatment that the patient actually received

Supplementary Figure 6:

Standardized mean difference (SMD) plot. Blue squares represent the SMDs before weighting with inverse probability of treatment weighting (IPTW), while yellow diamonds show the SMDs after weighting. The constructed propensity score successfully balanced all variables below an SMD of 0.1, resulting in no significant differences between treatment groups. Abbreviations: HFNC – high flow nasal cannula, NIV – noninvasive ventilation, SOFA – sequential organ failure assessment, COPD – chronic obstructive pulmonary disease, PEEP – positive end-expiratory pressure, BMI – body mass index, ECMO – extracorporeal membrane oxygenation, IL-6 – interleukin-6, CRP – C-reactive protein

Supplementary Figure 7:

Overall survival and IAPA incidence displayed as competing events for the whole cohort (n=172). The 30-day survival estimate is 64.8% (95% CI: 57.2-71.5), shown by the blue curve, while the IAPA incidence estimate is 15.2% (95% CI: 10.4-21.8), shown by the red curve. Abbreviation: ICU – intensive care unit.

Supplementary Figure 8:

90-day ICU survival according to antifungal prophylaxis (long-term survival). A) Unadjusted analysis. B) IPTW-adjusted analysis. p-values were calculated using the log-rank test. A risk table was included only for the unadjusted analysis. ICU survival was estimated using Kaplan–Meier curves. Abbreviations: IAPA – influenza-associated pulmonary aspergillosis, AF – empirical antifungal treatment.

Supplementary Figure 9:

Line graph showing serum (solid lines) and BAL (dashed lines) galactomannan levels in three ICU patients over Days 1, 3, and 5. Optical density index values decline over time. Aspergillus PCR results shift from positive on Day 1 to negative by Day 5, indicating response to early empirical posaconazole treatment
